# Assessment of Self-report, Palpation, and Surface Electromyography Dataset During Isometric Muscle Contraction

**DOI:** 10.1038/s41597-024-03030-8

**Published:** 2024-02-15

**Authors:** Jihoon Lim, Lei Lu, Kusal Goonewardena, Jefferson Zhe Liu, Ying Tan

**Affiliations:** 1 Department of Mechanical Engineering, The University of Melbourne, Parkville, 3010 Australia; 2 Department of Engineering Science, University of Oxford, Oxford, OX1 2JD UK; 3 Department of Population Health Sciences, King’s College London, London, UK; 4 Elite Akademy Sports Medicine, Parkville, 3010 Australia

**Keywords:** Fatigue, Biomedical engineering

## Abstract

Measuring muscle fatigue involves assessing various components within the motor system. While subjective and sensor-based measures have been proposed, a comprehensive comparison of these assessment measures is currently lacking. This study aims to bridge this gap by utilizing three commonly used measures: participant self-reported perceived muscle fatigue scores, a sports physiotherapist’s manual palpation-based muscle tightness scores, and surface electromyography sensors. Compensatory muscle fatigue occurs when one muscle group becomes fatigued, leading to the involvement and subsequent fatigue of other muscles as they compensate for the workload. The evaluation of compensatory muscle fatigue focuses on nine different upper body muscles selected by the sports physiotherapist. With a cohort of 30 male subjects, this study provides a valuable dataset for researchers and healthcare practitioners in sports science, rehabilitation, and human performance. It enables the exploration and comparison of diverse methods for evaluating different muscles in isometric contraction.

## Background & Summary

Muscle fatigue, characterized by a decline in muscle performance and accompanied by feelings of weakness, tiredness, or exhaustion in the affected muscles, is a prevalent and non-specific symptom experienced by many individuals^1,2^. It can be associated with a range of health conditions, including muscle strain, chronic fatigue syndrome (CFS), and overtraining syndrome, which can result from the accumulation of untreated muscle fatigue over time^2,3^. Therefore, monitoring muscle fatigue plays a crucial role in providing timely intervention for these conditions. However, detecting and measuring muscle fatigue poses significant challenges due to its complex nature. It involves multiple components of the motor system, including mechanisms within the brain and spinal cord, peripheral nerves, neuromuscular junction, excitation-contraction coupling, and force generation^4^. The intricate interplay of these components makes it difficult to isolate and quantify specific parameters related to muscle fatigue.

Despite these challenges, researchers and clinicians have proposed various measures to assess muscle fatigue, which can be categorized into two classes. The first class comprises subjective measures that rely on self-reported data from participants or subjective assessments conducted by clinicians or physiologists. Examples of subjective measures include self-administered questionnaires^5^, the Borg CR-10 scale^6^, and a palpation-based muscle tightness scale often used in clinical or research settings^7,8^; where the palpation-based scale involves the physiotherapist manually assessing the muscle and providing a subjective rating based on the level of muscle tension and tightness. These measures provide valuable insights into the subjective experience of muscle fatigue and its impact on individuals. However, it is important to note that these subjective measures generally have limitations in terms of reliability. For instance, the Borg CR-10 scale may provide unstable estimations due to its subjective nature^9^.

The second class consists of objective measures that utilize various sensors and quantitative methods to assess muscle fatigue. These objective measures provide more quantitative and precise insights into muscle fatigue. They include blood tests, electromyography (EMG), or surface EMG (sEMG) to measure electrical activities of muscles, force measurements using dynamometers or force plates, and other sensor-based technologies like accelerometers or wearable devices. For example, motion sensors have been employed to assess the perceived muscle fatigue on coordination during endurance running^10^, and accelerators were found efficient to monitor fatigue during intermittent exercise^11^. These objective measures contribute to a more comprehensive and objective evaluation of muscle fatigue. Among these sensors, sEMG sensors have been widely used due to their simplicity of use. The sEMG signal can provide valuable information by decomposing sEMG signals to extract information and neural activation^12,13^ and characterize muscle fatigue through changes in signal indicators^14–18^. Some commonly used sEMG measures include the mean absolute value (MAV), root mean square (RMS), mean frequency (MNF), and median frequency (MDF), nonlinear variables such as percentage of recurrence and determinism by recurrence quantification analysis (RQA)^19,20^ among others. For instance, sEMG signal was utilized to assess muscle fatigue during a forward head and rounded shoulder sitting posture^21^; it has been used to evaluate the effects of fatigue on muscle synergies for baseball players^22^; another study also used sEMG signal to detect localized muscle fatigue for track and field athletes^23^.

Understanding muscle fatigue is a challenging task since it is influenced by various factors such as psychological, physiological, and sociological factors^24,25^. A combination of subjective and objective measures can provide more robust insights into muscle fatigue analysis and studies are comparing the subjective and objective measures of muscle fatigue^26–32^. However, these studies have several shortcomings: (i) a limited number of sensors attached to a restricted area of muscle parts, for example, only one sEMG sensor on the right trapezius muscle is used^30^. (ii) a small sample size, and (iii) a lack of sEMG signals, which play an important role in understanding muscle activities. For example, pressure andoxygenated hemoglobin levels were measured using a pressure sensor^28^, as well as vertical jump levels without employing the sEMG sensor^29^. Therefore, the goal of this paper is to enable a comprehensive understanding of muscle fatigue by presenting data collected through three measures: (1) participant’s self-reported perceived muscle fatigue rank, (2) muscle tightness evaluations conducted by an experienced sports physiotherapist employing manual palpation-based techniques, and (3) extracted feature from sEMG signal measurements. These measures have been selected for their widespread usage and ability to offer valuable insights into the assessment of muscle fatigue. It is highlighted that the experiments were instructed by an experienced physiotherapist with over thirty years of physiotherapy services in sports, providing a high level of expertise in the clinical field. To assess and measure compensatory muscle fatigue, the sports physiotherapist selected nine distinct upper body muscles for evaluation. These specific muscles were chosen based on their involvement in the task and their potential to contribute to muscle fatigue.

The study comprises three datasets aimed at understanding the occurrence and consequences of compensatory muscle fatigue. Firstly, participants were asked to report the top three most fatigued muscles at the conclusion of the experiments which is perceived muscle fatigue, providing valuable subjective insights into their individual experiences. It is known in clinical practice that tight muscles are often associated with inefficient function and a higher susceptibility to fatigue^33,34^. Thus, in the second step of the experiment, a sports physiotherapist proficiently assigned a continuous muscle tightness score to each of the nine selected muscles. Manual palpation by sports physiotherapists, as conducted in this study, presents a potential measure for assessing muscle fatigue. Additionally, at the conclusion of the experiments, the physiotherapist identified and scored the top three muscles exhibiting the highest tightness, utilizing his expertise and manual palpation-based techniques. Lastly, sEMG sensors were used to record muscle activity from the nine selected muscles, under the guidance of the experienced sports physiotherapist.

To the best of our knowledge, this work represents the first comprehensive set of data, shedding light on the occurrence and dynamics of compensatory muscle fatigue during specific movements. This dataset offers several advantages for further research and analysis. Firstly, it serves as a valuable resource for exploring and comparing diverse methods used to assess muscle fatigue. Researchers can utilize this dataset to gain insights into the strengths and limitations of different approaches, advancing the field of muscle fatigue assessment. Secondly, the dataset provides a unique opportunity to investigate compensatory muscle fatigue in a controlled and standardized manner. By analyzing the data, researchers can gain valuable insights into the mechanisms and dynamics of this phenomenon, deepening our understanding of how muscles interact and adapt during physical tasks. Thirdly, this dataset can serve as a benchmark for future studies in the field. It provides a reference point for replicating and validating findings, ensuring the reliability and reproducibility of research in the area of compensatory muscle fatigue. Furthermore, the dataset can inspire the development of new methodologies and approaches for studying and quantifying muscle fatigue.

Overall, this dataset holds immense potential for advancing our knowledge of muscle fatigue and its implications in various fields, including sports science, biomechanics, rehabilitation, and human performance.

## Methods

### Participants

In this study, we recruited thirty healthy male participants without any history of neurological or muscular pathology from Australia. The experiments were performed between August 2022 and December 2022. Among the participants, 28 had a dominant right hand, and 2 had a dominant left hand. Each participant held the dumbbell using their dominant hand. Out of the thirty participants, 29 completed the entire experimental protocol, while one individual was suspended in the middle of the process due to muscle fatigue.

Prior to the experiment, all subjects were instructed to disclose any medical conditions or medications to the sports physiotherapist. To minimize the impact of prior physical activities, participants were advised not to exercise or engage in heavy lifting for at least three hours before the start of the session. It was also emphasized that participants perform proper warm-up exercises to reduce the risk of injury.

Before commencing the experiment, each participant provided information regarding their dominant hand, height, weight, and age^35^ (see Table 1). Detailed information about the experimental protocol was provided to all participants, and they were required to sign a consent form prior to their involvement in the study. The study protocol was approved by the Human Research Ethics Committee of the University of Melbourne (ID: 1954575).

**Table 1:** Anthropometric characteristics of thirty male study participants.

	Age	Dominant Hand	Height [*m*]	Weight [*kg*]	BMI [*kg*/*m*^2^]
Subject 01	29	Right	1.76	82	26.5
Subject 02	27	Right	1.72	80	27.0
Subject 03	27	Right	1.80	71	21.9
Subject 04	27	Right	1.76	75	24.2
Subject 05	25	Right	1.75	73	23.8
Subject 06	31	Right	1.85	105	30.7
Subject 07	30	Right	1.84	72	21.3
Subject 08	30	Right	1.80	105	32.4
Subject 09	33	Right	1.82	76	22.9
Subject 10	25	Right	1.71	78	26.7
Subject 11	24	Right	1.77	100	31.9
Subject 12	24	Right	1.75	74	24.2
Subject 13	27	Right	1.75	75	24.5
Subject 14	25	Right	1.72	74	24.0
Subject 15	26	Right	1.78	88	27.8
Subject 16	25	Left	1.75	72	23.5
Subject 17	39	Right	1.81	70	21.4
Subject 18	28	Right	1.80	96	29.6
Subject 19	30	Right	1.77	73	23.3
Subject 20	25	Right	1.75	72	23.5
Subject 21	36	Right	1.79	83	25.9
Subject 22	27	Right	1.66	60	21.8
Subject 23	32	Right	1.72	85	28.7
Subject 24	33	Right	1.82	78	23.5
Subject 25	28	Right	1.65	77	28.3
Subject 26	31	Right	1.81	84	25.6
Subject 27	29	Right	1.68	75	26.6
Subject 28	27	Right	1.72	64	21.6
Subject 29	29	Left	1.70	70	24.2
Subject 30	31	Right	1.72	63	21.3

Body Mass Index (BMI) is calculated by dividing the weight in kilograms by the height in meters squared.

### Experimental setup

**Fig. 1:**
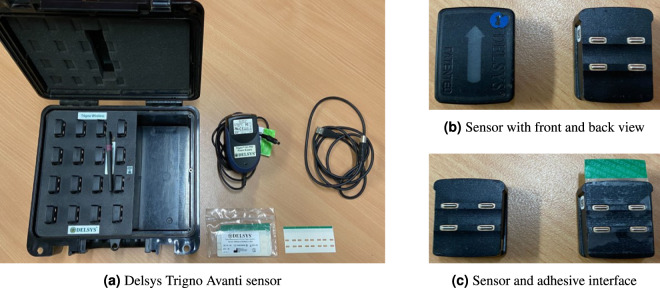
Delsys Trigno Avanti surface EMG sensor system.

In this study, commercially available Delsys Trigno Avanti sEMG sensors (Delsys Incorporations, USA) were used as shown in Fig. 1a. These sensors were attached to the skin using a customized double-sided adhesive interface without conductive paste or gels, and compressive pressure was applied to enhance the adhesive strength as shown in Fig. 1b,c. The adhesive tape is single-use only and medical-grade approved for dermatological applications. The use of the adhesive interface ensured an electrical connection between the sensor and the skin, minimizing noise from line interference.

However, it is important to note that the planar, flat, and rigid surface of commercial sEMG sensors makes them vulnerable to motion artifacts caused by relative motions^36^. These motion artifacts can contaminate the sEMG signals^37^. Additionally, the sensors may fall off from the curvilinear parts of the human skin, even with the tailor-made adhesive interface, during dynamic movements^38^ To address these limitations, sEMG signal recording in this study was conducted with participants in a static posture with isometric muscle contraction. This approach minimized the influence of motion artifacts and ensured the stability of the sensor placement throughout the experiment.

Based on the selected static posture with isometric muscle contraction, the experienced sports physiotherapist identified nine different locations of muscle groups on the upper limb and body. These specific muscle locations were carefully chosen, considering the potential sequential fatigue that might be triggered by this particular movement, in order to analyze compensatory muscle fatigue.

When using sEMG sensors, the placement of sensors on the skin plays a critical role in ensuring high-quality signals and reliable measurements. The electrode orientation refers to aligning the line connecting the two bipolar electrodes with the direction of the muscle fibers^39^.

Aligning the electrodes in the direction of the muscle fiber is important as muscle activity signals depend on this orientation. In addition, the sensor locations were carefully selected to avoid innervation zones, origin or insertion locations, and the edge of the muscle belly. Therefore, the sensors were positioned following the recommended location and orientation of the muscle fibers to enable accurate estimation of spectral parameters^39–41^.

Moreover, each sensor placement was isolated strategically from other muscles to minimize muscle crosstalk and interference. Even small displacements of the electrodes within a centimeter range had a huge impact on the signal, not ensuring reliable readings. Additionally, the selected sensor locations were chosen to have minimal hair, reducing the muscle crosstalk from surrounding muscles.

**Fig. 2:**
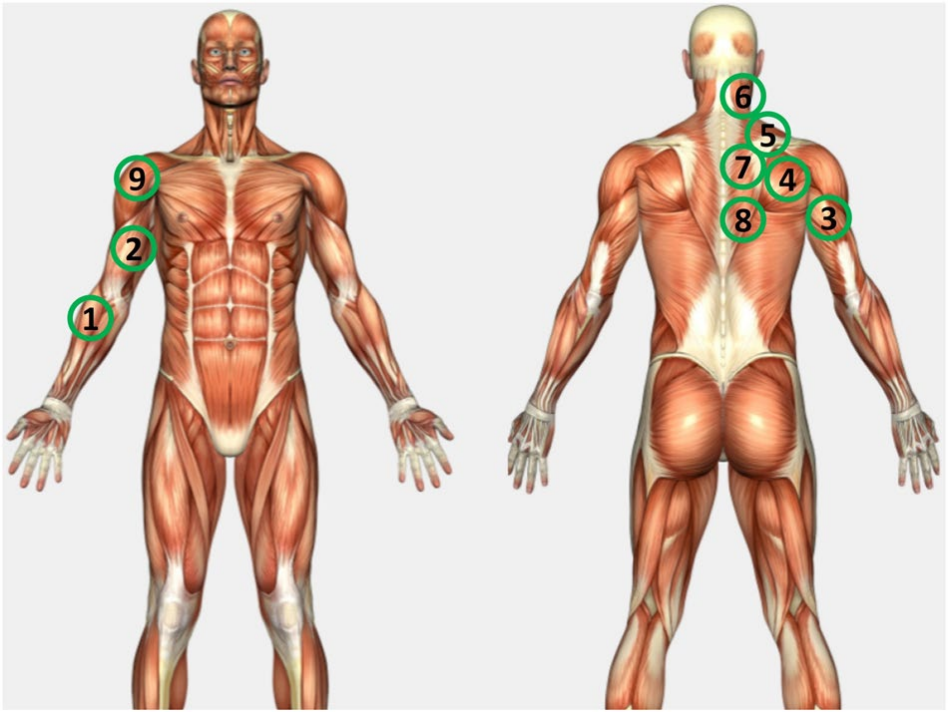
Sensor locations on nine different muscles: Brachioradialis (# 1 (BR)), Biceps Brachii (# 2 (BB)), Triceps Brachii (# 3 (TB)), Infraspinatus (# 4 (IS)), Upper Trapezius (# 5 (UT)), Paraspinal Cervical Spine (# 6 (PCS)), Middle Trapezius (# 7 (MT)), Lower Trapezius (# 8 (LT)), Anterior Deltoid (# 9 (AD)).

The placement of the nine wireless Delsys Trigno Avanti sEMG sensors on the upper body followed specific guidelines from the literature^39,40,42,43^, and was examined by a physiotherapist at our local sports center. Firstly, the six sensor placement locations (# 2 (BB), # 3 (TB), # 5 (UT), # 7 (MT), # 8 (LT), and # 9 (AD)) were marked with erasable markers based on the guidelines (*SENIAM; Surface Electromyography for the Non-Invasive Assessment of Muscles*) for the sEMG sensors. Additionally, three additional locations (# 1 (BR), # 4 (IS), and # 6 (PCS)) were selected based on recommendations from the sports physiotherapist. The muscle and sensor locations are described in Fig. 2 and detailed information about each sensor placement and orientation can be found in Table 2. In Fig. 2, muscle locations are indicated for right-handed participants.

**Table 2:** Muscle, EMG sensor location, and EMG sensor orientation^40,42,43^.

	Muscle	EMG Sensor Location	EMG Sensor Orientation
# 1	Brachioradialis (BR)	Electrode needs to be placed approximately 1/3 of the distance between the lateral epicondyle of the humerus and the styloid process of the radius	In the direction of the muscle fibers, which run longitudinally along the forearm
# 2	Biceps brachii (BB)	Electrodes need to be placed on the line between the medial acromion and the fossa cubit at 1/3 from the fossa cubit	In the direction of the line between the acromion and the fossa cubit
# 3	Triceps brachii (TB)	Electrodes need to be placed at 50% on the line between the posterior crista of the acromion and the olecranon at 2 finger widths medial to the line	In the direction of the line between the posterior crista of the acromion and the olecranon
# 4	Infraspinatus (IS)	Electrodes need to be placed approximately two fingerbreadths below the spine of the scapula, which is the bony ridge that runs along the posterior surface of the scapula	In a horizontal orientation, with the long axis of the sensor aligned with the direction of the muscle fibers, which run diagonally across the posterior aspect of the scapula
# 5	Upper Trapezius (UT)	Electrodes need to be placed at 50% on the line from the acromion to the spine on vertebra C7	In the direction of the line between the acromion and the spine on vertebra C7
# 6	Paraspinal Cervical Spine (PCS)	Electrodes need to be placed at the level of the muscle belly, which is typically located 2–3 cm lateral to the midline of the spine	In a vertical orientation, with the long axis of the sensor aligned with the direction of the muscle fibers, which run parallel to the spine
# 7	Middle Trapezius (MT)	Electrodes need to be placed at 50% between the medial border of the scapula and the spine, at the level of T3	In the direction of the line between T5 and the acromion
# 8	Lower Trapezius (LT)	Electrodes need to be placed at 2/3 on the line from the trigonum spinea to the 8th thoracic vertebra	In the direction of the line between T8 and the acromion
# 9	Anterior Deltoid (AD)	Electrodes need to be placed at one finger width distal and anterior to the acromion	The direction of the line between the acromion and the thumb

The entire experimental procedure was conducted under the supervision of an experienced sports physiologist, ensuring the precise positioning of sensors and accurate measurement of muscle activities. The presence of the experienced physiotherapist guaranteed consistent sensor placement for each participant, mitigating the potential effects of crosstalk and other sources of interference.

### Experimental protocol

Prior to commencing the experiments, several precautions were taken to ensure optimal sEMG signal quality. The sports physiotherapist provided a detailed explanation of the experimental protocol and addressed any questions or concerns from the participants, ensuring their understanding and cooperation throughout the process. To prepare for the attachment of sensors, participants were instructed to remove their upper garments, allowing for direct contact between the sensors and the skin. Then, in order to maintain hygiene and minimize contact impedance between the electrodes and the skin, the designated skin areas for sensor placement were thoroughly cleansed using antiseptic skin wash, effectively removing any surface residues. Subsequently, the skin was completely dried to ensure firm electrode-skin contact. Any electronic devices or accessories that had the potential to interfere with the signal quality were removed to ensure accurate and reliable measurements.

During the experiment, participants remained bare-bodied from the waist up, adopting an upright posture with their torso in a relaxed position. The attachment of sensors began by placing each sensor on the target muscles of the dominant upper limb and body, following the predetermined locations identified by the sports physiotherapist.

In this study, the participants underwent a single movement test, which consisted of two separate sessions: a preparatory session (Ses1) and a data collection session (Ses2) as shown in Table 3.

**Table 3:** Experimental Protocol.

	Experiment Procedure	Repetition	Duration [s]
Ses1: Preparatory session	Calibration of sensors	1	10
Recovery	Relax	1	30
Ses2: Data collection session	1) Muscle tightness measurements for 210 seconds with 30-second intervals	1	210
	2) sEMG data collection		
Rank assessment	Self-reported and palpation-based method	1	60

#### Preparatory session (Ses1)

During the trial session of the dumbbell frontal raise, each lasting 10 seconds, the sEMG signals of each muscle were assessed. The recording session immediately followed, with the consecutive performance of the exercise. Commercial sEMG sensors recorded the electrophysiological signals from nine different muscles, which were displayed in real-time on a computer screen. Participants were instructed to avoid any movement in elbow and wrist flexion/extension during the exercise. After the trial session, participants had a 30-second rest period to ensure muscle readiness and minimize potential muscle fatigue before the data collection session.

For the calibration of sensors, we checked the display if the signal reading from all nine sensors was correct or wrong. The real-time Signal Quality Monitor panel was used to check whether noise interference or not good adhesion of the sensor on the skin was found. If the signal quality is not good which is outside of the green area on the gauge panel or the Bluetooth connection is not stable, reattachment was conducted.

**Fig. 3:**
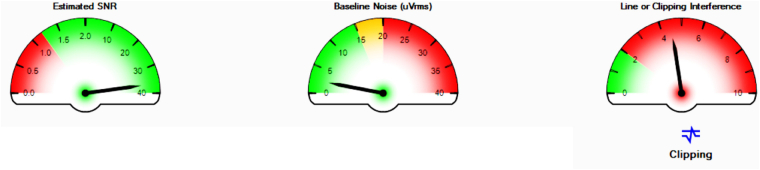
Delsys sensor signal quality check.

In the experiments, when all factors were pointing to green status, we initiated the data collection process which confirmed the acquisition of a high-quality sEMG signal. If any of these factors fell within the yellow or red areas on the scale, for example, shown in Fig. 3, calibration procedures such as checking for baseline noise on the display panel, adjusting the sensor’s position, and skin preparation were done again. These actions were taken to address any signal quality issues and ensure the reliability of the recorded data.

#### Data collection session (Ses2)

**Fig. 4:**
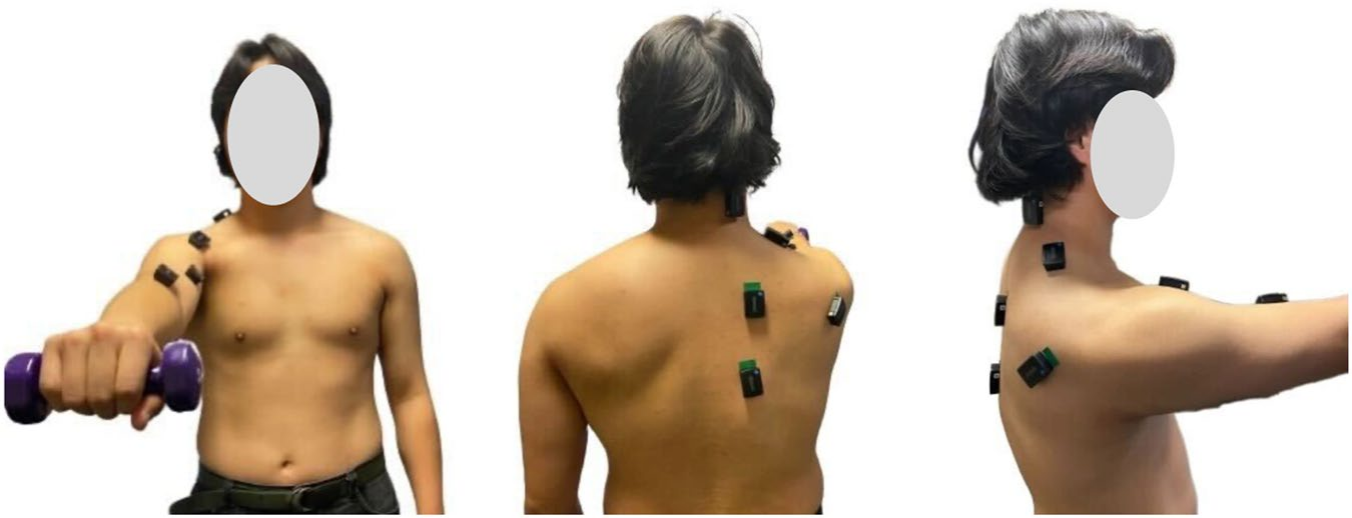
Nine sensors attached on the upper body (Front-view, Back-view, side-view).

In the data collection session, the nine sensors were attached to designated muscle locations on the subject’s upper body according to the experimental protocol. The subject assumed a comfortable and stable standing position with feet shoulder-width apart. As a starting position, the subject held a 1 kg dumbbell in their dominant hand, allowing the arm to hang straight down by their side with a pronated grip which means that the palm facing down. Slowly, the dominant arm was raised forward until it reached a peak position which is a parallel position to the ground while maintaining a straight arm and neutral wrist. The sports physiotherapist visually monitored the arm position and at the top of the lift, the dominant arm should be at or slightly below shoulder level, which is 90 to 180 degrees of shoulder flexion, with 180 degrees indicating a fully extended arm. Participants were instructed to maintain a stationary posture throughout the experiment while holding the 1 kg dumbbell in their dominant hand as shown in Fig. 4.

The figures presented in this study show muscle placements and locations as indicated for a right-handed participant. For left-handed individuals, the sensors were attached in the opposite orientation to prevent any potential confusion in the interpretation of the results. This approach was taken to ensure consistency in the sensor placement and to cater to both right-handed and left-handed participants.

During the exercise, a sports physiotherapist conducted regular assessments of muscle tightness using a palpation-based approach. At intervals of every 30 seconds, the sports physiotherapist evaluated the participant’s muscle tightness using a muscle tightness 4-point ordinal scale ranging from 0 to 3^44^. This continuous assessment of muscle tightness was carried out throughout the entire exercise duration of 210 seconds, allowing for the observation of muscle tightness progression over time. The chosen exercise duration aimed to replicate sustained effort and enable the monitoring of muscle fatigue development^45^. At the end of the exercise, the participant slowly lowered their arm back to the starting position and rested for 60 seconds. Participants were advised to discontinue the exercise if they experienced muscle fatigue to the extent that they could no longer maintain the required posture. Following the experiment, all sensors were detached, and both the skin and sensors were gently cleansed using an antiseptic skin wash to ensure hygiene and prepare for subsequent sessions or analyses.

Following the completion of the exercise, each participant was requested to rank the top three locations among the nine monitored muscles that they subjectively perceived as the most fatigued. In the case of muscles located on the backside, namely # 4 (IS), # 5 (UT), # 6 (PCS), # 7 (MT), and # 8 (LT), which were challenging to distinguish individually for muscle fatigue. Therefore, a sports physiotherapist assisted by pointing to each muscle to help participants identify and rank the muscle that was causing perceived muscle fatigue. This self-reported ranking provided participants with the opportunity to express their personal assessment of the muscles that they felt experienced the highest degree of fatigue during the exercise and aimed to collect subjective feedback on perceived muscle fatigue. Concurrently, the sports physiotherapist also ranked the top three muscles that exhibited the most significant signs of tightness. In this study, the palpation-based muscle tightness score data included the continuous assessment conducted by the sports physiotherapist as well as the ranking of the muscles at the end of the experiment based on his assessment.

The total duration of the experiment, including both preparation and data collection, was estimated to be approximately 20 minutes. The preparation phase accounted for approximately 10 minutes. The remaining time was dedicated to the actual data collection session, during which the participants performed the designated exercise while their muscle activity was recorded.

## Data Collection and Processing

In the data collection and processing phase of the study, three categories of data were recorded: (1) self-reported perceived muscle fatigue data, (2) palpation-based muscle tightness score data, and (3) sEMG signal data.

All collected data underwent comprehensive processing and analysis. Custom-written scripts in Matlab (The MathWorks Inc., US) were created and employed for the data processing tasks. They provided essential tools to process the data in a systematic manner, allowing for the extraction of meaningful insights and outcomes.

### Self-reported perceived muscle fatigue data

As part of the experimental protocol, participants were instructed to provide self-reports on the top three muscle sites among a predefined selection of nine muscles where they perceived muscle fatigue. This self-report assessment relied on participants’ subjective feelings and perceptions of muscle fatigue in these specific muscle sites. By capturing participants’ personal experiences and sensations, this self-reporting approach added a valuable subjective dimension to the evaluation of perceived muscle fatigue during the experiment. Table 4 presents two examples of self-reported data, demonstrating the format of the data. In this table, *sf*_1_, *sf*_2_, and *sf*_3_ represent the top three muscles that showed the most perceived fatigue for each subject, respectively.

**Table 4:** Two examples (Subject 09 and Subject 15) demonstrating the ranking of the top three muscles that showed the most perceived fatigue based on participants’ self-reported data.

	*sf*_1_	*sf*_2_	*sf*_3_
Subject 09	# 9 (AD)	# 8 (LT)	# 1 (BR)
Subject 15	# 9 (AD)	# 2 (BB)	# 1 (BR)

### Physiotherapist’s palpation-based muscle tightness score data

In addition, an experienced sports physiotherapist with expertise in palpation conducted a categorical scoring system by subjective examination of soft tissue tightness during the experiment. This examination yielded two sets of data. The first set, referred to as *Data 1*, consists of continuous assessments made by the sports physiotherapist throughout the experiment. The second set, referred to as *Data 2*, represents the physiotherapist’s ranking of muscle tightness at the end of the experiment for each participant.

The physiotherapist’s palpation aimed to assess muscle tightness rather than directly evaluating ‘fatigue’. It is a clinical understanding that tight muscles tend to function inefficiently and are more prone to experiencing fatigue^33,34^. Therefore, the assessment aimed to identify the top three muscles with the highest levels of tightness, as determined by the sports physiotherapist. This approach was used to indirectly gauge the potential impact of muscle tightness on muscle fatigue.

To illustrate the format of the collected data, Table 5 provides two examples of *Data 1*, showcasing the sports physiotherapist’s palpation-based scores of muscle tightness for the nine monitored muscles.

**Table 5:** Two examples (Subject 09 and Subject 15) of *Data 1* for sports physiotherapist’s palpation-based assessment of muscle tightness during the 210-second experiment with 30-second intervals.

(a) Subject 09								
Subject 09	0 s	30 s	60 s	90 s	120 s	150 s	180 s	210 s
# 1 (BR)	1	3	3	3	3	3	3	3
# 2 (BB)	2	2	2	3	3	3	3	3
# 3 (TB)	0	1	1	1	1	1	2	2
# 4 (IS)	2	2	3	3	3	3	3	3
# 5 (UT)	2	3	3	3	3	3	3	3
# 6 (PCS)	1	2	2	2	2	2	2	2
# 7 (MT)	2	2	3	3	3	3	3	3
# 8 (LT)	1	2	2	2	3	3	3	3
# 9 (AD)	2	3	3	3	3	3	3	3
**(b) Subject 15**								
**Subject 15**	**0 s**	**30 s**	**60 s**	**90 s**	**120 s**	**150 s**	**180 s**	**210 s**
# 1 (BR)	1	3	3	3	3	3	3	3
# 2 (BB)	2	2	2	3	3	3	3	3
# 3 (TB)	0	1	1	1	1	1	2	2
# 4 (IS)	2	2	3	3	3	3	3	3
# 5 (UT)	2	3	3	3	3	3	3	3
# 6 (PCS)	1	2	2	2	2	2	2	2
# 7 (MT)	2	2	3	3	3	3	3	3
# 8 (LT)	1	2	2	2	3	3	3	3
# 9 (AD)	2	3	3	3	3	3	3	3

Numbers in the table are manual palpation-based muscle tightness scores ranging from 0 to 3 measured by a sports physiotherapist.

Table 6 provides two examples of *Data 2*, demonstrating the final assessment of the sports physiotherapist. In this table, *pf*_1_, *pf*_2_, and *pf*_3_ represent the top three muscles with the highest level of muscle tightness from the sports physiotherapist, respectively.

**Table 6:** Two examples (Subject 09 and Subject 15) demonstrating the ranking of the top three muscles with the highest level of muscle tightness from the sports physiotherapist (*Data 2*).

	*pf*_1_	*pf*_2_	*pf*_3_
Subject 09	# 9 (AD)	# 5 (UT)	# 7 (MT)
Subject 15	# 5 (UT)	# 9 (AD)	# 7 (MT)

### sEMG data

**Fig. 5:**
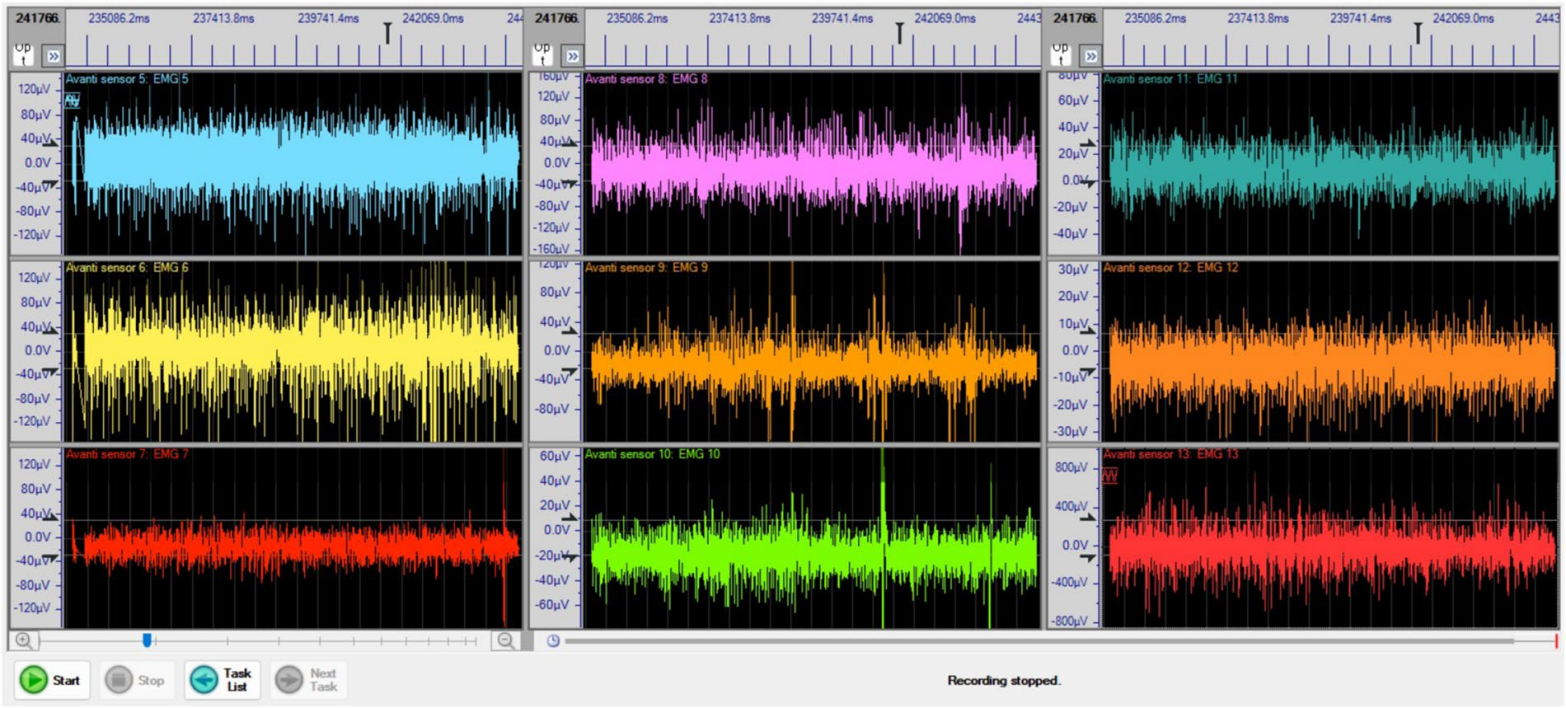
sEMG signals acquisition using EMGworks Acquisition 4.8.0 software for nine different muscles on upper body (brachioradialis (# 1 (BR)), biceps brachii (# 2 (BB)), triceps brachii (# 3 (TB)), infraspinatus (# 4 (IS)), upper trapezius (# 5 (UT)), paraspinal cervical spine (# 6 (PCS)), mid trapezius (# 7 (MT)), lower trapezius (# 8 (LT)), anterior deltoid (# 9 (AD))).

A commercially available Delsys Trigno Avanti sensor system was utilized for the detection and measurement of muscle activities in nine specific upper body muscles of each subject. An example of sEMG data is shown in Fig. 5. All nine sEMG sensors were synchronized on time. During data collection, the sEMG signals were acquired using the EMGworks Acquisition 4.8.0 software and recorded at a sampling rate of 2148 Hz. The sEMG sensor transmitted the data in real-time to the *Lenovo Thinkbook* laptop (*Intel*(*R*) *Core i*7–1165*G*7 @ 2.80 *GHz*, 16*GB RAM*) wirelessly which was connected to the base station via a USB cable. All the data collected from the software is exported to an Excel spreadsheet for further analysis. The demonstration of sEMG signals from Subject 19 for 9 different muscle locations on the screen of the EMGworks Acquisition system is shown in Figure 5.

The sEMG signal from the commercial sensor refers to the sEMG signal acquired after it has been amplified and subjected to bandpass filtering. This filtering process involves the use of analog components equipped with filters. Specifically, the Butterworth 2nd-order high-pass filter and a 4th-order low-pass filter were used to ensure the entire frequency spectrum within the specified range is captured^46^. Additionally, a 20 Hz high-pass filter is integrated to reduce the motion artifact which distorts the underlying physiological signal^47^. These integrated design features demonstrate the good signal quality of sEMG signals in this study.

**Fig. 6:**
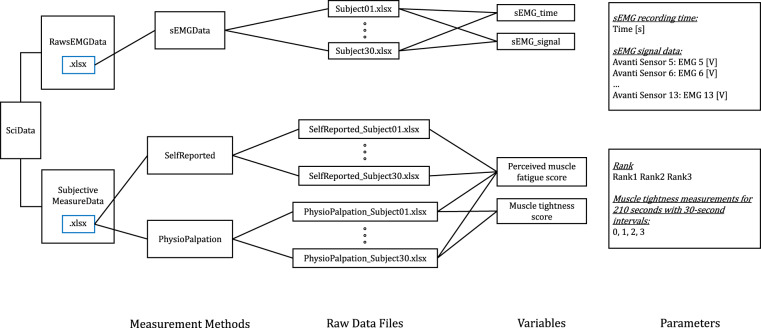
Schematic representation of the provided dataset. The *SciData* dataset is organized into two types of files: .xlsx and .mat files. The combination of .xlsx files in the *SciData* dataset allows for a comprehensive representation of the data.

## Data Records

The dataset accompanying this paper is available for download on *figshare*^48^. It is provided under an open license, allowing users to freely utilize the data for any purpose. In Fig. 6, the schematic representation of the dataset organization illustrates how the files in the dataset are structured and organized. The figure shows the hierarchical arrangement of files and variables and parameters for each file.

**Fig. 7:**
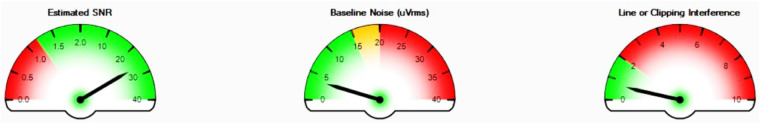
Technical validation of sEMG sensor signal quality.

The raw data from the subjective measurements (self-reported perceived muscle fatigue scores and physiotherapist’s palpation-based muscle tightness measurement scores) are stored in individual files encoded in .xlsx format. Each file corresponds to a specific measurement and contains the relevant data collected during the experiment. These files serve as a comprehensive record of the subjective assessments performed by the participants and the sports physiotherapist. Furthermore, the sEMG data using 9 different sensors on corresponding muscles recorded from each participant are stored in separate files encoded in .xlsx format. These files contain a set of sEMG sensor variables that collectively constitute the participant’s data. Each variable represents a specific aspect of the recorded data and contributes to a comprehensive understanding of the participant’s measurements.

### Self-reported perceived muscle fatigue rank data

Subjective measurement data of participants’ self-reported perceived muscle fatigue rank were summarized in Excel spreadsheet format (e.g., *SelfReported_Subject01.xlsx*).

- *Subject*: Each data file is named according to the participant number, which is an integer ranging from 1 to 30.
- *Sensor*: Sensor 01 - Sensor 09 corresponds to the muscle part described in Fig. 2.
- *Self-reported perceived muscle fatigue Rank 1, Rank 2, Rank 3*: The data records for self-reported perceived muscle fatigue rank 1, 2, and 3 include information on the participants’ subjective assessment of their muscle fatigue levels. Each record specifies the participant number, the rank of perceived muscle fatigue (1, 2, or 3), and the corresponding muscle site. These records provide insights into the participants’ individual perceptions of muscle fatigue and contribute to understanding the subjective experience of fatigue during the experimental sessions.

### Physiotherapist’s palpation-based muscle tightness rank data

Sports physiotherapist’s palpation-based assessment of muscle tightness during the 210-second experiment with 30-second intervals and final assessment of muscle tightness was summarized in Excel spreadsheet format (e.g., *PhysioPalpation_Subject01.xlsx*).

- *Subject*: Each data file is named according to the participant number, which is an integer ranging from 1 to 30.
- *Sensor*: Sensor 01 - Sensor 09 corresponds to the muscle parts described in Fig. 2.
- *Muscle tightness measurements for 210* *seconds with 30-second intervals*: The subjective data records for each participant include the physiotherapist’s palpation-based measurements taken at 0 which is the starting point and 30-second intervals for a total of 8 times across nine muscle locations.
- *Sports physiotherapist’s palpation-based muscle tightness Rank 1, Rank 2, Rank 3*: Followed by the muscle tightness measurement with 30-second intervals, the data records for sports physiotherapist-assessed muscle tightness rank 1, 2, and 3 contain the evaluations conducted by the physiotherapist. Each record includes the participant number, the rank of muscle tightness assigned by the physiotherapist (1, 2, or 3), and the associated muscle location. These records reflect the expert judgment of the physiotherapist regarding the severity and localization of muscle tightness, providing valuable assessments of muscle condition during the experimental sessions.

### sEMG data

Raw data contains sEMG data for all subjects with nine muscles. The sEMG time and signal data were collected via a Bluetooth module and an in-house data acquisition (DAQ) system. The recorded data was stored in Excel Spreadsheets in .xlsx format, with each participant’s data saved in a separate file (e.g., *Subject01.xlsx*).

- *Time*: The sEMG raw time data consists of the time series measurements recorded from the sEMG sensors. These sensors captured the electrical activity generated by the muscles during the experimental sessions. Each data entry in the time series corresponds to a specific time point. The sEMG raw time data is stored in an Excel spreadsheet (.xlsx) using *Time [s]* format.
- *sEMG signal*: The sEMG signal data contains the amplitude of the electrical signals recorded by the sEMG sensors. These signals represent the muscular electrical activity and provide insights into the muscle’s activation levels during the experimental sessions. Each entry in the signal data corresponds to a specific time point, reflecting the magnitude of the electrical activity at that particular moment. The sEMG signal data is stored in an Excel spreadsheet (.xlsx) using *Avanti sensor 5: EMG.A 5 [V]* format.

In the sensor configuration, each sensor consists of four electrodes. The upper two electrodes are differential sEMG pairs, and the lower two electrodes are stabilizing references. It allows the sensor to quickly respond to disturbances detected on the skin surface which reduces the impact of potential noise sources^49^. Additionally, bar electrodes composed of 99.9% silver with a 10 mm inter-electrode distance (IED) were positioned to minimize the crosstalk from surrounding muscles effectively and ensure the good signal quality^50^.

## Technical Validation

This section contains the sEMG sensor configuration and acquisition. In the context of sEMG sensor placement for signal assessments, the guideline in the appendix “Interpretation of Muscle and Signal Quality Assessments” was used^39^. This guideline specifically offers criteria for assessing the quality of signals for sensor placements on the most superficial muscles during isometric contractions. To ensure the reliability of the recorded sEMG signals, the assessment considered four criteria with scores for each muscle. These criteria included the assessment of the signal quality based on its amplitude above the background noise level, the electrode placement to avoid innervation zones, the fidelity of the recorded signal to the natural propagation of muscle activity, and the feasibility of motor unit identification. Each muscle was scored based on how well these criteria were met. Following the guideline, seven muscles (# 1 (BR), # 2 (BB), # 3 (TB), # 5 (UT), # 7 (MT), # 8 (LT), # 9 (AD)) achieved the full score of 6 points, while one muscle (# 4 (IS)) received 5 points. Since the assessment guideline focused on only 43 muscles in the trunk, upper limb, and lower limb, the paraspinal muscle located on the cervical spine (# 6 (PCS)), situated in the neck region, was not included in the scoring system. Therefore, in our study, this metric system with scores validated the setup of the experiment, particularly regarding the careful selection of muscle locations and resulting high-quality sEMG recordings. Additionally, muscle tissue exhibits anisotropic properties, which emphasize the importance of aligning the detection surfaces of the electrodes with the orientation of the muscle fibers. To ensure accurate electrode placement, we collaborated with a sports physiotherapist, informed by relevant literature^39^

To guarantee the functionality of the integrated sEMG sensor and to confirm the high quality of the sEMG data collected, various metrics describing the sEMG acquisition process were taken into account. These metrics and their detailed descriptions can be found in the guideline^46^.

- *Input source impedance*: Input impedance refers to the resistance to current flow into each input terminal of an amplifier, and it varies with frequency. When dealing with dry skin, the input impedance at the interface between the skin and the detection surface can range from thousands to millions of ohms. Maximizing the input impedance of the differential amplifier is important to avoid signal loss or distortion due to input loading. It allows the accurate capture of electrode voltages without disruption. Moreover, amplifiers with high input impedance help minimize contamination from unwanted power line interference. This consideration ensures reliable sEMG signal recording without causing issues in the differential amplifier.
- *Differential amplifier gain*: The primary function of an amplifier is to take a weak electric signal originating from the body and amplify its amplitude to make it suitable for recording and display on electronic devices. In this study, a commercial standalone sEMG sensor, Delsys Trigno, with a gain of 1000 which is a gain value well within the accepted range was used^51^. This high gain significantly improved the signal-to-noise ratio (SNR) of the sEMG signal and made it highly resilient to noise and interference.
- *Common-mode rejection ratio (CMRR)*: The utilization of bipolar electrode arrangements is common with a differential amplifier, which effectively eliminates signals common to both electrodes. Typically, the common mode voltage, which is the signal common to both electrodes, is larger than the sEMG signal. The CMRR quantifies the differential amplifier’s accuracy in subtracting these common signals. Therefore, a high CMRR is essential to distinguish the sEMG signals from the background noise effectively. The Delsys commercial sensor utilized in this study shows a CMRR value exceeding 80 dB, which assures excellent signal quality^52^.
- *sEMG Bandwidth*: Frequency band of sEMG between 20 and 450 Hz. A 4th-order Butterworth band-pass filter was used to achieve an effective frequency range of sEMG signals between 20 Hz and 450 Hz, and a 2nd-order Butterworth band-stop filter with cut-off frequencies 49 Hz and 51 Hz was used to remove power frequency noise^47^.
- *Inter-electrode distance (IED)*: The size of electrodes and the space between them highly affect the sEMG signal. Larger detection areas and greater inter-electrode distances (IED) result in greater amplitude of the sEMG signal detection. However, these dimensions should not be too large to avoid picking up the crosstalk interference from neighboring muscles. Delsys sensors maintain a 10 mm IED, effectively reducing crosstalk while preserving sEMG signal amplitude^49^. This fixed IED ensures consistency and repeatability in experiments that maintain data quality and the integrity of sEMG signal acquisition.
- *Motion artifact*: Motion artifact is typically induced by relative movement of the sEMG sensor in relation to the skin. It is an interference on the electrode-skin interfaces and contaminates the signal quality. It becomes especially challenging when dealing with dynamic muscle contractions or rapid body movements. To ensure accurate contact and stable recording in our experiment, we conducted isometric contractions without rapid body movements. This static posture approach allowed us to maintain reliable contact between the sensor and the skin, minimizing unwanted noise during data recording.

Moreover, the Delsys EMGworks Acquisition software incorporates a real-time Signal Quality Monitor tool which provides continuous feedback on the sensor’s signal quality as shown in Fig. 7. This tool continuously monitors the signal quality of each sensor and provides visual feedback during the experiment. It assesses the estimated SNR within the range of 0–40, baseline noise ranging from 0–40 uVrms, and line or clipping interference ranging from 0–10, in real-time. Acceptable signal quality is indicated by an SNR greater than 1.2, baseline noise below 15 uVrms, and minimal line interference below 2, which is indicated by a green area from the gauge panel. This real-time monitoring system offers a dynamic way to ensure the quality of the recorded data throughout the experiment. Various factors related to signal quality and noise have also been considered and verified, affirming that the experiment’s signal quality meets acceptable standards^53^.

- *Signal-to-noise ratio (SNR)*: SNR is one of the most important quality measures of sEMG signal^54^. It quantifies the ratio between the sEMG signal recorded during muscle contraction and the baseline noise when the muscle is at rest. A higher SNR value indicates a more robust ability to reliably discriminate and extract sEMG data from unwanted noise.
- *sEMG baseline noise*: To ensure the quality of an sEMG signal, it is important to establish the baseline noise of the system. According to the EMGWorks software, Delsys sEMG systems typically exhibit a baseline noise of less than 15 µV which is within an acceptable range of 10–20 µV peak-to-peak from the literature^50^. The quality of the skin-electrode interface significantly influences the level of baseline noise. Thus, before commencing data collection, we conducted a preparatory session to check the baseline noise within a range and ensured the contact between the electrodes and the skin.
- *Line interference noise*: Noise at frequencies of 50 or 60 Hz, originating from power lines, fluorescent lights, and various electrical devices, is a common source of interference in sEMG recordings. Advanced sensor technology with designed circuits for the Delsys sEMG sensor has effectively eliminated this issue.
- *Clipping*: Signal saturation is a type of distortion that occurs when a signal surpasses a certain threshold. This can happen due to sensor detachment or excessively high sEMG signal amplitudes. To maintain signal integrity, it is crucial to monitor any signal clipping to ensure that the sEMG sensor and reference electrode are properly attached and connected. If required, adjustments can be made by reducing the gain or repositioning the sEMG sensor to lower the signal level, as recommended in the literature^55^. In our study, a gain of 1000 is used, which is an appropriate value for enhancing surface sEMG signals, and it is in an acceptable range while avoiding clipping.

Furthermore, three different performance metrics were calculated to check the signal quality. The drop in power density ratio (DPR) indicates whether the signal power spectrum is adequately peaked in the sEMG power spectrum’s frequency range. The power spectrum deformation (PSD) measures the effect of disturbances of the spectrum of a signal with a power spectrum larger than 20 Hz^56^. From Table 7, both DPR and PSD values showed that the Delsys surface EMG sensors have adequate peaking and are immune to high-frequency noise. Based on the signal quality analysis, these factors ensured that the signal quality maintained a high level of signal quality throughout the experiment.

**Table 7:** Signal quality analysis (Subject 19).

	# 1 (BR)	# 2 (BB)	# 3 (TB)	# 4 (IS)	# 5 (UT)	# 6 (PCS)	# 7 (MT)	# 8 (LT)	# 9 (AD)
SNR value	47.49	42.49	31.67	48.65	44.99	36.06	48.33	50.34	45.94
DPR value	40.36	42.82	28.77	45.60	38.03	37.84	45.29	46.12	42.47
PSD value	1.119	1.146	1.310	1.157	1.202	1.243	1.366	1.155	1.175

**Fig. 8:**
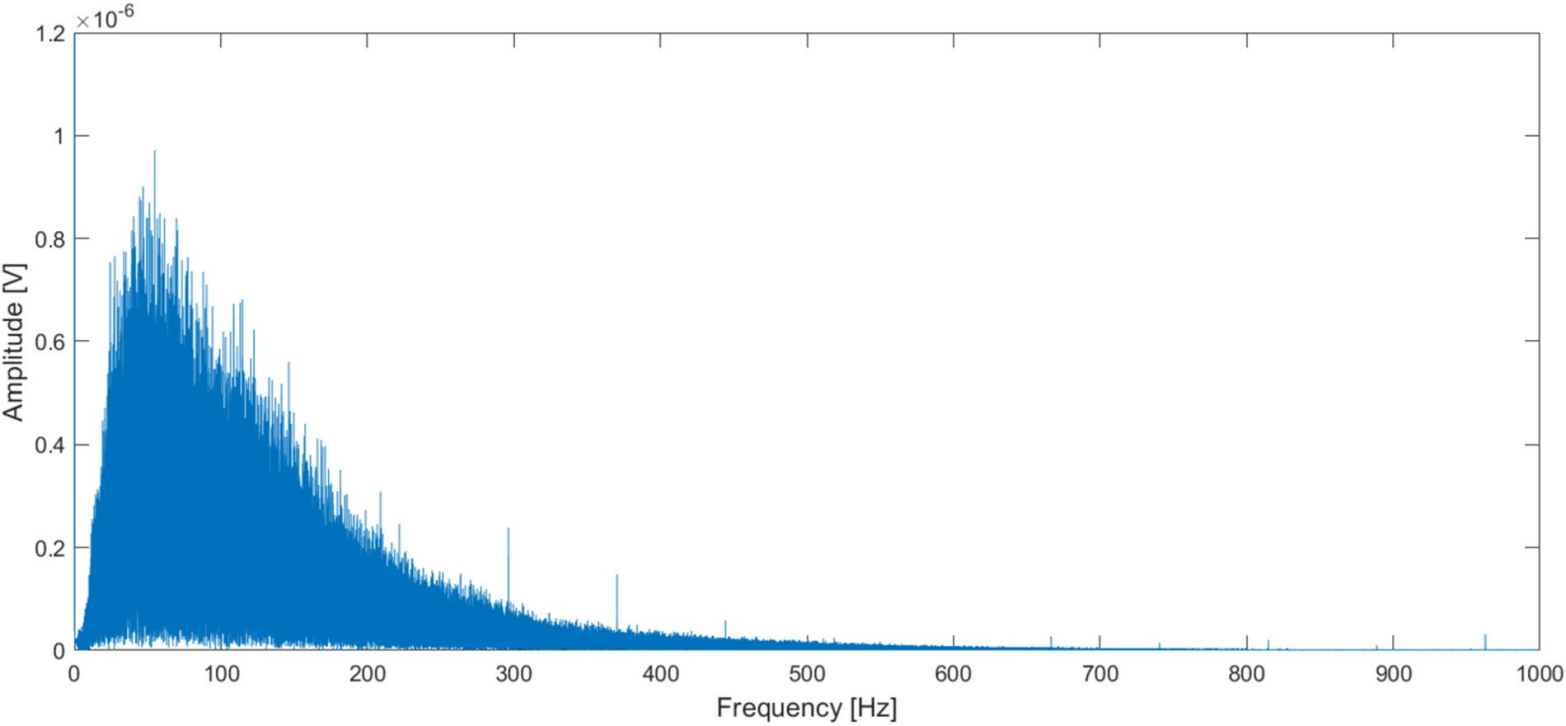
Frequency spectrum of the sEMG signal detected during an isometric contraction (Subject 19).

Subsequently, this section validates the sEMG measurements. One fundamental mathematical technique for analyzing signals is the Fourier Transform, which can deconstruct any signal into a series of sine waves with varying frequencies. We first check the frequency range of sEMG signals. It is known that the frequency range of sEMG signals is in [20 Hz, 450 Hz]^57,58^. We have checked the frequency range of all sEMG signals by using a fast Fourier transform (FFT) on sEMG signals. It has been verified that all sEMG signals measured stayed in this range, validating the sEMG signals collected from commercial sEMG sensors in this work. A visualization of the power distribution provides a comprehensive measure of how different frequencies impact the sEMG signal and an example of the frequency spectrum of an sEMG signal is shown in Fig. 8.

**Fig. 9:**
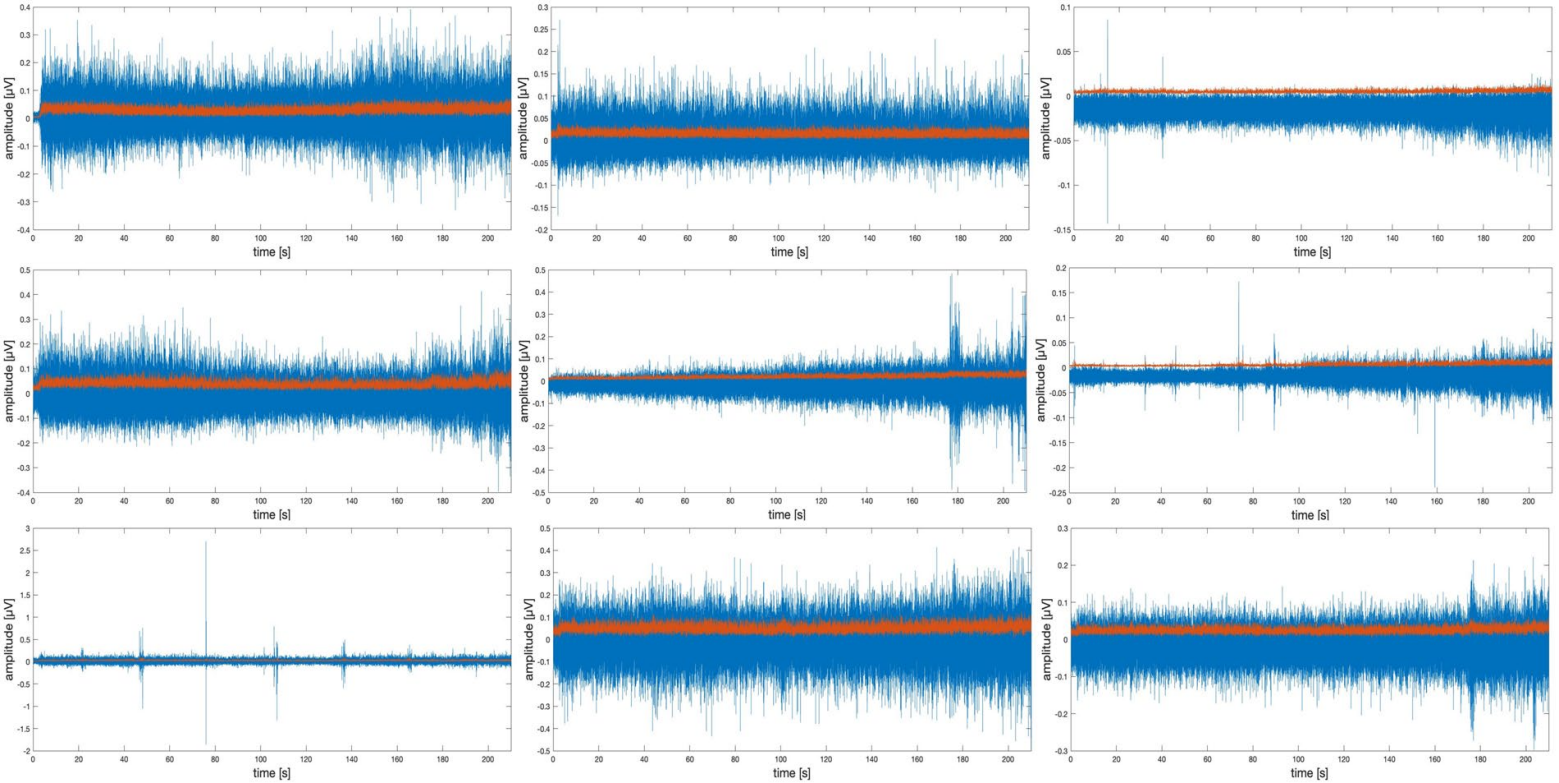
sEMG signal (blue) and pre-processed sEMG signal (red) for nine different muscles on the upper body (Subject 19, Brachioradialis (# 1 (BR)), Biceps Brachii (# 2 (BB)), Triceps Brachii (# 3 (TB)), Infraspinatus (# 4 (IS)), Upper Trapezius (# 5 (UT)), Paraspinal Cervical Spine (# 6 (PCS)), Middle Trapezius (# 7 (MT)), Lower Trapezius (# 8 (LT)), Anterior Deltoid (# 9 (AD))).

Then, signal processing techniques were used to reduce the contaminated noises^55^ and extract the features of the sEMG sensors^59^. The signal processing of sEMG signals was performed using custom-written Matlab scripts and the process included signal acquisition and pre-processing. The sEMG signal was sampled at a rate of 2148 Hz. The signal was then filtered by a digital bandpass filter with a passband between 20 Hz and 450 Hz based on the FFT analysis ensuring that no critical information is lost during signal acquisition^54^. The filtered signal was then rectified with full wave rectification and used for envelope analysis. The filtered signal is shown in Fig. 9.

Fatigue is a complex and widespread phenomenon that comes in various forms. It can be categorized as pathological or non-pathological, physical or mental, and can be evaluated subjectively or objectively. Various techniques have been employed to measure fatigue and energy levels. Some methods aim to gauge the impact of fatigue, such as reduced performance, while others aim to pinpoint the origins of fatigue, like muscle dysfunction.

The definitions of muscle fatigue are diverse, and they haven’t been definitively linked to concrete objective measures. This doesn’t undermine the value of both subjective and objective measures of fatigue but highlights the complexity of this phenomenon. While subjective measures of perceived muscle fatigue and objective measures using sEMG sensors are widely employed, the complexity of muscle fatigue persists. In addition to these conventional approaches, the palpation-based technique can be a possible measure that is linked to muscle fatigue. This technique introduces a tactile dimension, providing an alternative means to assess and understand muscle fatigue beyond the established subjective and objective measures.

Even though the integration of these measures remains unclear, both subjective and objective measurements are taken into account in the context of muscle fatigue, as they hold significance in assessing health and quality of life. In future research, it is crucial to bridge the gap between subjective and objective measures by considering multiple factors and conducting calibration studies. Additionally, there is a need for further investigations using hand-held dynamometers, experiments with heavier weights, and longer durations to enhance our understanding of compensatory muscle fatigue.

## Usage Notes

To use the provided code, you need to have Matlab installed, preferably version R2021b or higher. You can load the Matlab script file *SciDataEMG.m*, which is available in the provided link, for data processing and analysis. The dataset is categorized into three sub-groups: *SubGroup1.mat* comprises data from Subject 01 to Subject 10, *SubGroup2.mat* contains data for Subject 11 to Subject 20, and *SubGroup3.mat* includes data for Subject 21 to Subject 30. Then, select the relevant sub-group mat file based on the subject and muscle of interest, and specify the desired *subject_id* and *muscle_id*. For instance, if you wish to analyze muscle # 9 (PCS) of Subject 09, load *SubGroup1.mat*, and assign *subject_id* = 9 and *muscle_id* = 9. Executing these selections will generate the following plots: (1) sEMG signal plot and (2) sEMG signal and pre-processed sEMG signal plot.

## Data Availability

The custom-written code used for data acquisition and analysis in this paper can be downloaded from *figshare*^60^. The provided files contain the necessary scripts and functions for data acquisition and signal processing. • *readme.pdf* with instructions about loading the dataset, running the code, and code execution. • *SciDataEMG* contains: - Code (*SciDataEMG.m*) - The .mat files in the SciData dataset (*SubGroup1.mat*, *SubGroup2.mat*, *SubGroup3.mat*) contain summarized or processed data, which can be loaded into Matlab for further analysis and visualization. To facilitate data management and analysis, the data from all thirty participants were consolidated into a summarized format using Matlab. The raw sEMG time and signal data for each subgroup of participants were saved in a .mat file (e.g., *SciData/RawEMGData/MatlabData/SubGroup1.mat*) for computational efficiency since the dataset of 30 subjects is too large. These files are commonly used for efficient processing and analysis using Matlab functions and tools. - Results (*P_9_M_9 sEMG signal.png*, *P_9_M_9 pre-processed sEMG signal.png* which are plotted results for representative subject example from the code (Subject 09).
